# Real-world insights into the management of hemophilia A in Italy: treatment patterns and healthcare resource utilization

**DOI:** 10.1007/s44313-024-00034-6

**Published:** 2024-10-08

**Authors:** Valentina Perrone, Melania Leogrande, Maria Cappuccilli, Luca Degli Esposti

**Affiliations:** CliCon S.r.l. Società Benefit, Health, Economics & Outcomes Research, Bologna, Italy

**Keywords:** Healthcare resource utilization, Hemophilia A, Monoclonal antibodies, Plasma-derived FVIII, Treatment patterns

## Abstract

**Purpose:**

This real-world analysis described the Hemophilia A (HA) population in Italy, evaluating drug utilization and consumption of factor VIII (FVIII) products of patients under prophylaxis and on-demand therapy.

**Methods:**

From Jan-2017 to Jun-2022, male patients with HA were identified through prescriptions of FVIII products [extended half-life FVIII, standard half-life recombinant FVIII, and plasma-derived FVIII (EHL FVIII, SHL rFVIII, and pdFVIII, respectively)], or emicizumab or FVIII plus von Willebrand factor or HA-related hospitalization using administrative flows of Italian healthcare entities. Patients on treatment with FVIII products during 2021–2022 were stratified by treatment regimen (prophylaxis/on-demand). The mean annual consumption expressed in International Units (IU) of EHL FVIII and SHL FVIII in patients treated during 2021–2022 having at least 12-month follow-up were assessed.

**Results:**

Among included HA patients, 145 (39.5%) received EHL FVIII and 222 (60.5%) SHL FVIII. Of 165 patients on prophylaxis, 105 (64%) received an EHL FVIII and 60 (36%) an SHL FVIII. The mean annual consumption of FVIII was 336,700 IU (median 319,000 IU) for EHL FVIII and 440,267 IU (median 360,500 IU) for SHL FVIII. Specifically, for patients on EHL FVIII, the most common drugs were efmoroctocog alfa (*N* = 51) and damoctocog alfa pegol (*N* = 50), followed by turoctocog alfa pegol (*N* = 25) and rurioctocog alfa pegol (*N* = 19). Of 702 HA patients initially treated with FVIII products, 74 (10.5%) switched to emicizumab during follow-up.

**Conclusion:**

These findings revealed an extensive use of EHL FVIII products, suggesting growing efforts from clinicians to optimize prophylactic strategies and achieve better bleeding protection.

**Supplementary Information:**

The online version contains supplementary material available at 10.1007/s44313-024-00034-6.

## Introduction

Hemophilia A (HA) is a rare X-linked recessive hemorrhagic hereditary disease that arises from a congenital deficit of factor VIII (FVIII), resulting in prolonged and excessive bleeding, either spontaneous or secondary to trauma [1, 2]. Affecting approximately 1 in 5000 male births worldwide, HA is among the most commonly reported bleeding disorders [3]. HA has three levels of severity—mild, moderate, and severe—determined by the amount of clotting factor in an individual's blood. Mild hemophilia is characterized by clotting factor activity greater than 5% to 40% of normal, moderate hemophilia is presented with 1–5% of the normal amount, and severe hemophilia manifests when the clotting factor is less than 1% [4, 5].

In Italy, according to the 2022 report of the National Registry of Congenital Coagulopathies, 2,944 patients with HA were registered in 2020, of which 1,299 were considered to be affected by a severe form [6, 7]. This condition poses significant challenges, particularly in subjects with severe bleeding phenotype, demanding meticulous management to prevent complications. About two-thirds of patients who are diagnosed with hemophilia have other family members with the disorder [8, 9]. In the remaining cases, HA is typically identified through laboratory tests for abnormal bleeding in individuals with no significant family bleeding history [2, 10].

Replacement of FVIII has been used for decades in HA patients either to control bleeding, including during and after surgery, and to induce immune tolerance in those who develop alloantibodies able to inhibit FVIII coagulant activity [11].

In the 1950s, fresh frozen plasma was used as the first replacement therapy in patients with hemophilia, followed by cryoprecipitates during the 1960s, and then plasma-derived lyophilized FVIII in the 1970s [9]. Nevertheless, the high rates of infections transmitted with infusion therapy, including HIV during the 1980s, motivated researchers to develop safer plasma-derived products [9]. The cloning of the FVIII gene in 1984 was the starting point for the successive production of recombinant human FVIII (rFVIII) in 1992 [12]. This transformative development also paved the way for a shift from merely treating episodic bleeding events (on-demand treatments) to the implementation of prophylaxis regimens as a preventive measure [13, 14], resulting in increased life expectancy and better management of spontaneous bleeding of joints and muscles that represented a major cause of arthropathy and disability of HA patients [13].

In the past decade, there have been further remarkable therapeutic advances, including the availability of coagulation factors with an extended half-life, allowing for more prolonged intervals between treatments [14–16]. Emicizumab, a monoclonal antibody and FVIII mimetic antibody, was introduced in clinical practice in 2017 and represents one of the few licensed non‐replacement therapies for HA treatment worldwide [17–19]. Emicizumab acts by bridging activated factor IX and factor X, thus restoring the function of missing activated FVIII in HA [20].

Despite these indisputable progresses in the treatment of hemophilia, there are still limitations, issues and unmet clinical needs. In this context, this real-world analysis was conducted to describe the population with HA in Italy in terms of demographic and clinical characteristics of patients, with stratification between patients on prophylaxis and on-demand, and to evaluate how patients are managed in the clinical practice in terms of drug utilization, consumption of FVIII and consumption of resources.

## Materials and methods

### Study design and data source

An observational retrospective analysis was conducted on the administrative flows of a pool of Italian healthcare entities, from the regions Lombardy, Veneto, Liguria, Lazio, Abruzzo, Molise, Puglia, Campania, and Sicily, covering nearly 12 million health-assisted citizens and with data available from January 2009 to June 2023. Administrative databases store data on healthcare resources reimbursed by the Italian National Health Service (INHS). For the analysis purposes, data were retrieved from the following databases: beneficiaries’ database, including patients’ demographic data; pharmaceuticals database, for information on drug prescriptions, that is, the anatomical therapeutic code (ATC), prescription date, and number of packages; hospitalization database, for data on hospital admissions, that is, the date of admission, primary and secondary diagnosis identified by International Classification of Diseases, Ninth Revision, Clinical Modification (ICD-9-CM), and Diagnosis Related Group (DRG); outpatient specialist service database for information on specialist visits, diagnostic and laboratory tests with type and date of provision.

To ensure patients’ privacy, a univocal numeric code (Patient ID) was assigned to each subject. This code also allows the electronic linkage between the databases. All the results are provided in aggregated form, so they cannot be attributable to individual patients, either directly or indirectly. The study was approved by the local Ethics Committees of the healthcare units involved (the names of the Ethics Committees, protocol authorization codes, and dates of approval are provided in Supplementary Table S1). Along with the Data Privacy Guarantor Authority pronouncement, General Authorization for personal data treatment for scientific research purposes—no. 9/2014, informed consents were waived, as it was not possible to obtain them for organizational reasons.

### Study population

From January 2017 and June 2022, the analysis included only male patients who met at least one of the following criteria (proxy of HA diagnosis): (i) at least one prescription of coagulation FVIII products (ATC code: B02BD02); OR (ii) at least one prescription of emicizumab (ATC code: B02BX06); OR (iii) at least one hospitalization for HA (ICD-9-CM: 286.0); OR (iv) at least one prescription of coagulation FVIII and von Willebrand factor (ATC codes: B02BD06 or B02BD02 + B02BD10) and no hospitalization for von Willebrand’s disease (ICD-9-CM code 286.4). In detail, coagulation FVIII products included: (i) extended half-life FVIII (EHL FVIII), that is, efmoroctocog alfa, rurioctocog alfa pegol, turoctocog alfa pegol, damoctocog alfa pegol; (ii) standard half-life recombinant FVIII (SHL rFVIII), that is, included octocog alfa, lonoctogoc alfa, turoctocog alfa, simoctocg alfa, moroctocog alfa; and (iii) plasma-derived FVIII (pdFVIII), that is, human coagulation FVIII/Von Willebrand factor.

The index date was established as the date of the first prescription for drugs associated with HA within the inclusion period. In cases where patients were included solely based on hospitalization, the index date was identified as the date of admission. The characterization period covered a minimum of 12 months before the index date, ensuring that all patients had at least one year of available data before this point. The follow-up comprised all the available periods after the index date, ensuring that each patient had a data availability period of at least one year following the index date. Female subjects, patients without continuous inclusion during the study period (e.g., those who moved to another region), or those with less than 12 months of data availability before and after the index date were excluded from the analysis. Recombinant FVIIa (ATC code: B02BD08) or FVIII inhibitor bypassing activity (ATC code B02BD03), in addition to the above-mentioned treatments, were not included in the analysis in order to exclude patients with HA and inhibitors.

For all the patients included in the analysis, age at index date with a distribution organized by age ranges (0–5, 6–11, 12–17, 18–39, 40–59, 60–69, and ≥ 70 years) were recorded. The comorbidity profile was investigated in the 12 months before the index date by the Charlson Comorbidity Index. This scoring system assigns a weight to each of 19 concomitant diseases (a score of 0 indicates no comorbid conditions, while higher scores indicate a greater level of comorbidity) [21]. The most common comorbidities known to be frequently associated with HA [22] were searched in the characterization period before index date, namely acute coronary syndrome, cardiac dysrhythmias, cerebrovascular disease, atherosclerosis and aneurysm, and other peripheral vascular disease, atrial fibrillation, hypertension, dyslipidemia, arterial or venous embolism and thrombosis, osteoporosis, arthropathy, HIV (human immunodeficiency virus), HCV (hepatitis C virus), and HBV (hepatitis B virus). Criteria used for identifying the comorbidities in the database are listed in Supplementary Table S2.

### Treatment patterns for HA

The presence of the following treatments was considered for the management of HA and evaluated during the study period: coagulation FVIII (ATC code: B02BD02); von Willebrand factor and coagulation FVIII in combination (ATC code: B02BD06); von Willebrand factor (ATC code: B02BD10) and emicizumab (ATC code: B02BX06).

Moreover, for all the included HA-treated patients, data on prescriptions for coagulation FVIII are categorized as follows: (i) EHL FVIII; (ii) SHL rFVIII; (iii) SHL pdFVIII; (iv) monoclonal antibodies (Emicizumab). Patients on treatment with FVIII products during 2021–2022 and with at least 12 months of follow-up were stratified by treatment regimen (prophylaxis or on-demand) considering the year following the date of the last FVIII treatment. The type of regimen was assessed by identifying FVIII international unit (IU) higher (prophylaxis) and lower (on-demand) with respect to defined thresholds set for specific age groups (pediatric, age 0–12; adult, age 13 +), as previously described in the literature [23]. Among the patients with HA diagnosis and treated at index date with an FVIII agent, any switch to treatment with emicizumab during at least one year of follow-up period was recorded.

Healthcare resource use (HCRU) was also analyzed, focusing on the consumption of EHL FVIII and SHL FVIII products in the patients treated during the years 2021–2022 and with at least 12 months of follow-up.

### Statistical analysis

Descriptive statistical analyses were conducted on continuous variables, expressed as mean ± standard deviation (SD), and categorical variables expressed as numbers and percentages. Along with the “Opinion 05/2014 on Anonymization Techniques” drafted by the “European Commission Article 29 Working Party”, the analyses on subgroups of ≤ 3 patients were not issuable (NI) for data privacy, as data might be potentially attributable to single individuals. All analyses have been conducted using STATA SE version 17.0 (StataCorp LLC, College Station, TX, USA).

## Results

### Characteristics of the study population

**Fig. 1:**
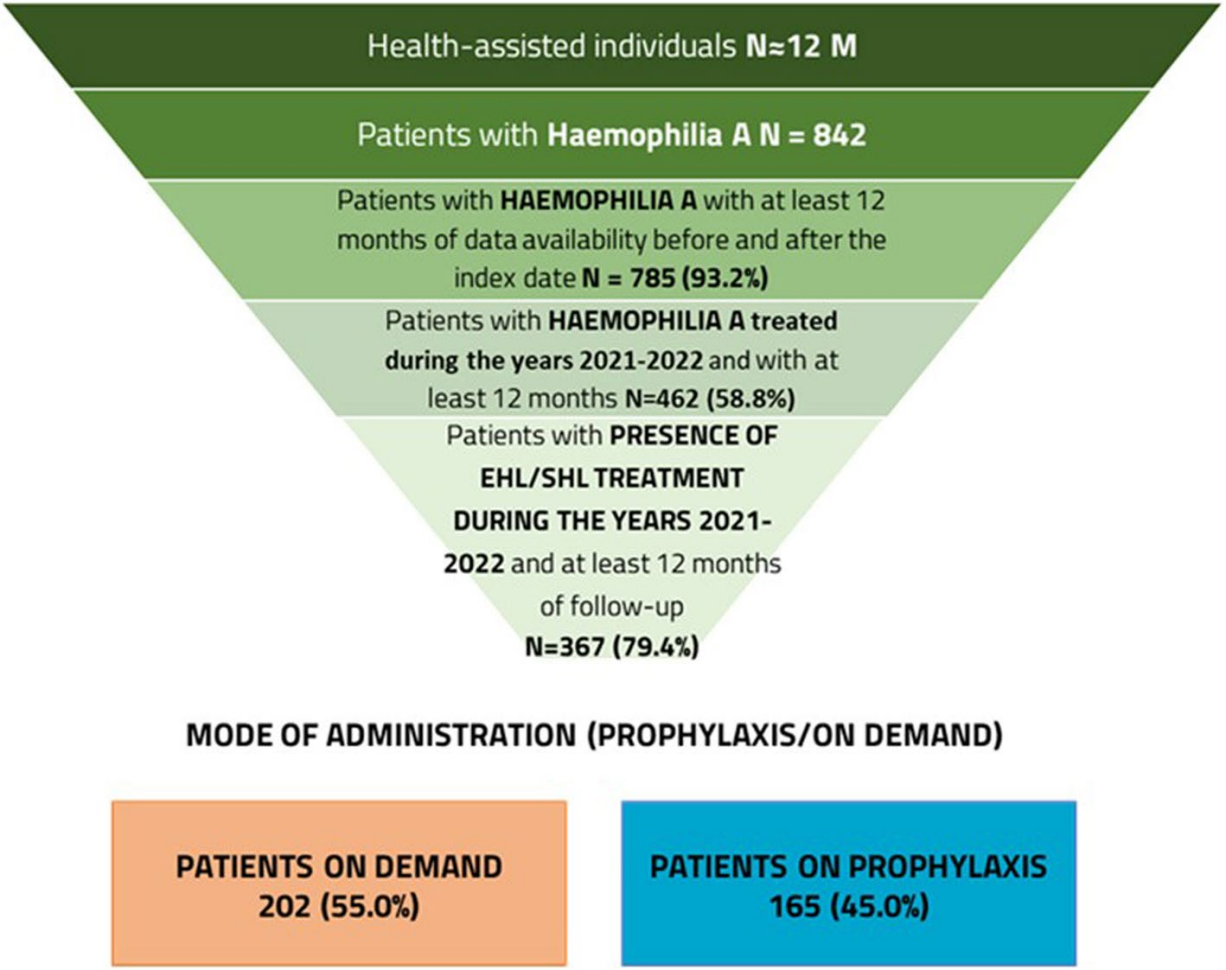
Scheme for patients’ selection

From a catchment area of around 12 million health-assisted individuals, 842 male patients with HA were identified in the administrative database from January 2017 to June 2022. Of them, only patients with at least 12 months of data availability before and after the index date were selected for the analysis (*N* = 785, 93.2%). Among these patients, a total of 462 individuals were under treatment for the disease during the years 2021–2022 and with at least 12 months of follow-up. Within the subgroup of patients treated with SHL/EHL FVIII therapies during 2021–2022 and at least 12 months of follow-up, 367 patients (79.4%) were selected for the analysis. Of them, 202 (55%) received on-demand therapy and 165 (45%) were on prophylaxis (Fig. 1).

The demographic and clinical characteristics in terms of comorbidities profile of patients with HA stratified by treatment regimen (on-demand and prophylaxis) are shown in Table 1. The mean age (± SD) at the index date is 36.7 (± 19.5) years for patients on-demand therapy and 35.7 (± 16.8) years for individuals on prophylaxis. In both cases, the most represented age classes are 18–39 and 40–59 years. The Charlson Comorbidity Index was low (less than 0.5), suggesting a mild comorbidity profile, as confirmed by the more extensive representation of patients in the score 0, regardless of treatment purpose. The most common concomitant condition was hypertension, which accounted for 20.3% of patients on-demand and 16.4% of individuals on prophylaxis, distantly followed by dyslipidemia (8.9%; 5.5%) and HCV infection (5.9%; 6.1%).

**Table 1:** Demographic and clinical characteristics of HA patients stratified by administration purpose (on-demand and prophylaxis)

	Patients on-demand (*N* = 202)	Patients on prophylaxis (*N* = 165)
**Demographic and clinical characteristics**		
Age at index-date (years), mean (± SD)	36.7 (± 19.5)	35.7 (± 16.8)
Age groups		
0–5 years, N (%)	8 (4.0%)	NR
6–11 years, N (%)	12 (5.9%)	10 (6.1%)
12–17 years, N (%)	25 (12.4%)	16 (9.7%)
18–39 years, N (%)	61 (30.2%)	70 (42.4%)
40–59 years, N (%)	65 (32.2%)	50 (30.3%)
60–69 years, N (%)	25 (5.0%)	15 (3.0%)
≥ 70 years, N (%)	6 (3.0%)	NR
Charlson index, mean (± SD)	0.4 (± 1.0)	0.3 (± 0.5)
Charlson index = 0, N (%)	141 (69.8%)	122 (73.9%)
Charlson index = 1, N (%)	48 (23.8%)	36 (21.8%)
Charlson index ≥ 2, N (%)	13 (6.4%)	7 (4.2%)
**Comorbidities**		
Hypertension, N (%)	41 (20.3%)	27 (16.4%)
Dyslipidemia, N (%)	18 (8.9%)	9 (5.5%)
HCV, N (%)	12 (5.9%)	10 (6.1%)
HIV, N (%)	8 (4.0%)	4 (2.4%)
HBV, N (%)	0 (0.0%)	0 (0.0%)
Arthropathy, N (%)	NR	NR
Cardiovascular disease, N (%)	0 (0.0%)	NR
Acute coronary syndrome, N (%)	0 (0.0%)	0 (0.0%)
Atrial fibrillation, N (%)	0 (0.0%)	0 (0.0%)
Arterial or venous embolism and thrombosis, N (%)	0 (0.0%)	0 (0.0%)
Osteoporosis, N (%)	0 (0.0%)	0 (0.0%)

*Abbreviations: HBV* Hepatitis B virus, *HCV* Hepatitis C virus, *HIV* Human immunodeficiency virus, *IU* International units*, NR* not reported for data privacy (< 3 patients), *SD* Standard deviation

### Consumption of FVIII products and HCRU

The analysis of the consumption of FVIII products, in patients with at least 12 months of follow-up treated during the years 2021–2022, revealed that 145 (39.5%) of them were prescribed with EHL FVIII therapy and 222 (60.5%) with an SHL FVIII therapy. Separate analyses were then conducted for patients on prophylaxis and those under on-demand therapy.

**Fig. 2:**
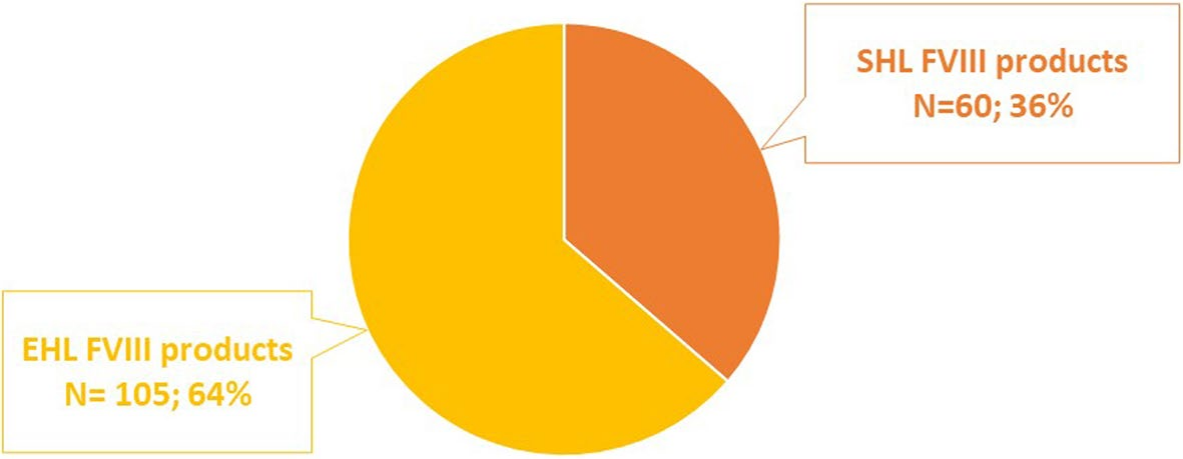
Distribution of prophylaxis treatments according to the different types of FVIII

Among the 165 patients on prophylaxis, 105 (64%) received an EHL FVIII product and 60 (36%) an SHL rFVIII (Fig. 2).

The mean annual consumption of FVIII, focused on patients on prophylaxis with at least 12 months of follow-up during the years 2021–2022, was 336,700 IU (median 319,000 IU) for EHL FVIII and 440,267 IU (median 360,500 IU) for SHL rFVIII. The detailed consumption for each EHL FVIII product is shown in Table 2. Specifically,, the most commonly prescribed drugs were efmoroctocog alfa (*N* = 51) and damoctocog alfa pegol (*N* = 50), followed by turoctocog alfa pegol (*N* = 25) and rurioctocog alfa pegol (*N* = 19).

**Table 2:** Annual consumption expressed in IU of FVIII among patients treated with EHL FVIII, overall (*N* = 145) and on prophylaxis (*N* = 105)

EHL FVIII	Overall patients (*N* = 145)	On prophylaxis, N (%) (*N* = 105)	Annual consumption in IU of FVIII (mean; median)
Efmoroctocog alfa	51	38 (74.5%)	290,579; 260,000
Damoctocog alfa pegol	50	40 (80.0%)	328,813; 303,500
Turoctocog alfa pegol	25	17 (68.0%)	440,647; 392,000
Rurioctocog alfa pegol	19	10 (52.6%)	366,800; 350,000

*Abbreviations: EHL* Extended half-life, *FVIII* Factor VIII, *IU* International units

A focused analysis was then conducted for adult HA population on prophylaxis (with at least 12 months of follow-up, *N* = 88) to investigate the monthly consumption of EHL FVIII during 2021–2022 (Table 3). Considering the consumption of EHL FVIII products on a monthly basis and on adult population only, damoctocog alfa pegol was the most comply prescribed (34.3%), followed by efmoroctocog alfa (25.7%). Efmoroctocog alfa was associated with the lowest observed consumption per month, either considering the overall adults patients on prophylaxis (25,941.4 IU), or after excluding those with high IU consumption 200% above the expected range (15,212.1 IU).

**Table 3:** Focus analysis on monthly consumption of EHL FVIII products on HA adults only (*N* = 88) on prophylaxis

	Adults HA patients only (*N* = 88) on prophylaxis			
	**Efmoroctocog alfa**	**Rurioctocog alfa pegol**	**Damoctocog alfa pegol**	**Turoctocog alfa pegol**
N (%)	27 (30.7%)	9 (10.2%)	36 (40.9%)	16 (18.2%)
Expected^a^				
Expected range of IU/month	**10,645.8**	**21,291.7**	**18,250**	**26,614.6**
***Observed***				
IU/month	**25,941.4**	**30,851.9**	**27,103**	**37,265.6**
N (%) patients > 25% expected range	27 (100.0%)	9 (100.0%)	36 (100.0%)	16 (100.0%)
N (%) patients > 50% expected range	27 (100.0%)	9 (100.0%)	36 (100.0%)	16 (100.0%)
N (%) patients > 75% expected range	27 (100.0%)	9 (100.0%)	36 (100.0%)	16 (100.0%)
N (%) patients > 100% expected range	27 (100.0%)	9 (100.0%)	36 (100.0%)	16 (100.0%)
N (%) patients > 125% expected range	23 (85.2%)	5 (55.6%)	27 (75.0%)	8 (50.0%)
N (%) patients > 150% expected range	20 (74.1%)	4 (44.4%)	15 (41.7%)	5 (31.3%)
N (%) patients > 175% expected range	19 (70.4%)	NI	8 (22.2%)	NI
N (%) patients > 200% expected range	16 (59.3%)	0 (0.0%)	NI	NI
*IU/month (excluded patients with high IU consumption, i.e.* > *200% expected range)*	***15,212.1***	***30,851.9***	***26,118.9***	***36,000***

*Abbreviations: EHL* Extended half-life, *FVIII* Factor VIII, *IU* International units, *NI* Not issuable (for data privacy: subgroups of ≤ 3 patients)

^a^“Expected” refers to the minimum value required to consider a patient in prophylaxis

Additionally, despite the limited number of patients, an investigation into FVIII consumption was extended to those treated with emicizumab. Of the 702 patients diagnosed with HA and initially treated with FVIII, 74 individuals (10.5%) switched to emicizumab during the follow-up period. The analysis of FVIII consumption was focused on this subgroup, comprising 46 patients who switched to emicizumab and had at least one year of follow-up data available. Among these 46 patients, 20 (43.5%) consistently received FVIII prescriptions throughout the entire follow-up period, with a mean (± SD) follow-up duration of 4.7 (± 1.2) years. Within this group, 10 patients (21.7%) had a prescription for FVIII in the first 3 months following the switch, while 19 (41.3%) had a prescription later than 3 months from the switch. Considering only 12 months of follow-up after the switch, 15 (32.6%) had at least one FVIII prescription, with a mean consumption of 77,333 IU (median 22,000 IU).

Table 4 describes the mean (± SD) annual number per patients of delivered HCRU among 462 HA patients treated during 2021–2022 with at least 12 months of follow-up, stratified by category of FVIII products. The number of yearly drug prescriptions of HA-related treatments was 9.2 (± 5.4) for patients on EHL FVIII, 7.6 (± 6.9) for patients on SHL rFVIII, 6.0 (± 5.5) for patients on SHL pdFVIII and 9.9 (± 4.3) for patients on emicizumab. The prescriptions of other drugs averaged 5.6 (± 10.0) in patients on EHL FVIII, 5.9 (± 10.5) in patients on SHL rFVIII, 6.5 (± 6.7) in patients on SHL pdFVIII and 3.1 (± 5.5) in patients on emicizumab. The number of outpatient specialist services provided was 3.1 (± 4.7) in patients on EHL FVIII, 2.3 (± 3.5) in patients on SHL rFVIII, 3.5 (± 4.5) in patients on SHL pdFVIII, and 2.5 (± 3.6) in patients on emicizumab. The number of annual hospitalizations per patient was 0.1 (± 0.5) in patients on EHL FVIII, 0.1 (± 0.4) in patients on SHL rFVIII, 0.2 (± 0.5) in patients on SHL pdFVIII, and 0.1 (± 0.3) in patients on emicizumab.

**Table 4:** Healthcare consumptions (expressed as number of provisions/services per patient) among HA patients treated during 2021–2022 and with at least 12 months of follow-up (*n* = 462), stratified by category of FVIII

Number per patient	Patients on EHL FVIII (*n* = 145)	Patients on SHL rFVIII (*n* = 222)	Patients on SHL pdFVIII (*n* = 37)	Patients on emicizumab (*n* = 58)
Prescriptions for HA, mean (± SD)	9.2 (± 5.4)	7.6 (± 6.9)	6.0 (± 5.5)	9.9 (± 4.3)
Other drug prescriptions, mean (± SD)	5.6 (± 10.0)	5.9 (± 10.5)	6.5 (± 6.7)	3.1 (± 5.5)
Outpatient specialist services, mean (± SD)	3.1 (± 4.7)	2.3 (± 3.5)	3.5 (± 4.5)	2.5 (± 3.6)
Hospitalizations, mean (± SD)	0.1 (± 0.5)	0.1 (± 0.4)	0.2 (± 0.5)	0.1 (± 0.3)

EHL FVIII includes efmoroctocog alfa, rurioctocog alfa pegol, turoctocog alfa pegol, damoctocog alfa pegol; SHL rFVIII includes octocog alfa, lonoctogoc alfa, turoctocog alfa, simoctocg alfa, moroctocog alfa; SHL pdFVIII include human coagulation FVIII/Von Willebrand factor

*Abbreviations: EHL* Extended half-life, *HA* Hemophilia A, *SD* Standard deviation, *rSHL FVIII* Standard half-life recombinant FVIII

## Discussion

The present analysis, conducted in a normal clinical practice setting, provided an up-to-date picture of HA management in Italy. The large amount of data extracted from the administrative databases could allow for increased knowledge of the therapeutic management of HA patients of all ages and adults only.

This comprehensive dataset, representing nearly 20% of the entire Italian population, facilitated the identification of 842 patients with HA from Jan 2017 to Jun 2022. Of them, 785 patients had a minimum of 12 months of data availability before and after the index date, forming the basis for our in-depth examination. Subsequently, focusing on the years 2021–2022, 367 patients who received EHL FVIII or SHL FVIII products and with had at least 12 months of follow-up were included in the analysis, allowing for a thorough evaluation based on the mode of FVIII administration, distinguishing between on-demand and prophylaxis. Regardless of administration mode, the HA population was relatively young (around 36 years of age), with patients predominantly concentrated in the 18 and 59 age range. These findings are consistent with a recent real-world evidence (RWE) study on Italian patients by Cortesi et al*.* who reported a mean (± SD) age of hemophilic patients of 37.2 (± 17.4) years [24]. The low CCI was suggestive of a mild comorbidity profile, but this is consistent with the average young age of the included patients.

In our population, the distribution of type of FVIII therapy revealed that around 40% of patients were treated with EHL FVIII products (of whom 72.4% were on prophylaxis), and the remaining 60% were treated with SHL FVIII products (of whom 27% were on prophylaxis). Moreover, the analysis of the mean consumptions of FVIII IU delivered to patients on prophylaxis therapy and according to all categories of coagulation FVIII showed that efmoroctocog alfa was the one associated with the lowest yearly consumption per patient among the various agents. This might be potentially attributed to its long half-life, requiring fewer injections for bleeding reduction during prophylaxis, as suggested by the results of the ASPIRE extension study, an open-label, non-randomized trial on 5-year follow-up, reporting that pediatric and adult HA patients, receiving efmoroctocog alfa showed low annualized bleeding rates (ABRs) and could be treated with long dosing intervals [24, 25].

A recent systematic literature review of RWE studies on efmoroctocog alfa treatment in HA patients in Europe corroborated the view that switching from a SHL rFVIII to prophylactic use of efmoroctocog alfa was a successful strategy to reduce FVIII concentrate consumption, maintaining low bleeding rates [26].

Data emerging from clinical trials and from RWE seem to suggest that EHL FVIII products might provide significant clinical and economic benefits either by decreasing therapy burden or increasing the level of bleeding protection [25, 26]. These trends were also noticed in our HA patients in prophylaxis, as about 4 out of 10 received an EHL FVIII product. In this regard, efmoroctocog alfa was the most commonly administrated of the EHL FVIII therapies, but it was also associated with the lowest annual IU consumption. This finding might be explained by the drug the half-life that is up to 1.8 times longer than that of conventional FVIII/rFVIII preparations [27]. The analysis conducted on adult HA population on prophylaxis confirmed that efmoroctocog alfa resulted in the lowest observed monthly consumption, although damoctocog alfa pegol was commonly prescribed in above one-third of HA adults on prophylaxis.

Taken together, these data generated from the real clinical practice in Italy suggest a shift in the treatment paradigm and increased awareness to focus on more protection from bleeding. The setting of hemophilia care has received significant benefits from the advent of novel EHL FVIII products that consent a lower frequency of infusions to achieve comparable or even higher trough/overall activity, over the previous standard regimens requiring every other day or three-times-per-week injection schedules [24].

The concurrent use of FVIII and emicizumab during the induction period has been previously observed in the scientific literature. An initial increase in FVIII administrations may be attributed, at least in part, to physicians prescribing FVIII for on-demand use to manage the risk of unexpected bleeding during the loading phase of emicizumab [28, 29]. Following the transition period, continued use of FVIII may be aimed at managing unexpected bleeding, enhancing prophylaxis for physical activity, and in the case of major or minor surgery. As generally recommended by the AICE (Italian Association of Hemophilia Centres) guidelines for all patients with severe HA on prophylaxis, in order to ensure adequate and timely treatment of intercurrent episodes, patients on prophylaxis with emicizumab should also be provided with a home supply of FVIII concentrate [30].

The main strength of this analysis lies in the large sample size generated from the real clinical practice of a sample corresponding to about 20% of the country’s population. This approach based on data from unselected study populations can facilitate the generalizability of the results, especially in the setting of rare diseases. However, there are some limitations to acknowledge. Despite the mentioned advantages in the use of administrative flows, there might be incompleteness or limited accuracy of certain information in the mentioned databases, like severity of comorbidities, severity of HA, or other potential confounding factors. For this reason, the analysis can only infer the prescription date, not the administration date. Moreover, the concomitant conditions included in the Charlson Comorbidity Index, and the comorbidities associated with HA were detected using drug prescriptions and hospitalizations as proxies of diagnosis. Therefore, the present analysis might not capture untreated or non-hospitalized comorbidities. Body weight is not traceable in the databases, and this missing information might have partly biased our assessment of FVIII product consumption (in IU). Moreover, in the current analysis, we only assessed the consumption EHL FVIII or SHL-rFVIII for patients in prophylaxis, while on-demand treatment and pdFVIII not considered. Lastly, some important variables that might potentially affect clinical outcomes, including joint health and lifestyle habits (i.e., physical activity, patient attitude towards medication, diet) are not traceable in the databases.

In conclusion, this real-world analysis conducted on HA confirmed the characteristic young age of patients, aligning with existing literature. The utilization of EHL FVIII products was higher compared to that found in clinical trial settings, possibly reflecting growing attention from clinicians on the concept of normalized hemostasis and better bleeding protection.

## Supplementary Information

Supplementary Material 1.

## Data Availability

All data used for the current study are available upon reasonable request next to CliCon s.r.l. which is the body entitled to data treatment and analysis by Local Health Units.

## Data availability

No datasets were generated or analysed during the current study.
