# Supplementary material for: Real-world insights into the management of hemophilia A in Italy: treatment patterns and healthcare resource utilization
Source: Blood Res. 2024 Oct 8;59(1):30. doi: 10.1007/s44313-024-00034-6 (PMC11461399; doi:10.1007/s44313-024-00034-6)
Supplement: Supplementary file 1 — Supplementary Material 1. [file 44313_2024_34_MOESM1_ESM.docx]

*SUPPLEMENTARY MATERIALS*

**Real-world insights into the management of** **Hemophilia A in Italy: treatment patterns and healthcare resource utilization**

**Table S1.** Authorization of the local Ethics Committee of the participating healthcare entities with protocol codes and approval dates.

| Authorization of the Ethics Committee of Bergamo “Comitato etico interprovinciale Area I” (protocol code PROGETTO STREAM, approval date 16/12/2022) |
| --- |
| Authorization of the Ethics Committee of Berica “Comitato Etico per le Sperimentazioni Cliniche (CESC) della Provincia di Vicenza” (protocol number 1627, approval date 28/10/2020) |
| Authorization of the Ethics Committee of Foggia “Comitato etico interprovinciale Area I” (protocol number 63/CE/20, approval date 3/12/2020) |
| Authorization of the Ethics Committee of Genova “Comitato Etico Regionale Liguria,” (protocol number 0179046/2020, approval date 14/06/2021) |
| Authorization of the Ethics Committee of Latina “Comitato Etico Lazio 2” (protocol number 0171576/2021, approval date 09/09/2021) |
| Authorization of the Ethics Committee of Molise Region “ASREM” (protocol number 101125, approval date 20/10/2020) |
| Authorization of the Ethics Committee of Napoli 3 “Comitato Etico Inter-aziendale Campania Sud” (protocol number 51, approval date 02/09/2020) |
| Authorization of the Ethics Committee of Palermo “Comitato Etico Palermo 1” (protocol number 02/2021, approval date 24/02/2021) |
| Authorization of the Ethics Committee of Pescara “Comitato Etico delle Province di Chieti e Pescara” (protocol number 07, approval date 18/03/2021) |
| Authorization of the Ethics Committee of Roma 3 “Comitato Etico Lazio 2” (protocol number 0031200/2021, approval date 10/02/2021) |
| Authorization of the Ethics Committee of Roma 4 “Comitato Etico Lazio 1” (protocol number 1079/CE Lazio 1, approval date 23/09/2020) |
| Authorization of the Ethics Committee of Roma 5 “Comitato Etico Lazio 1” (protocol number 1166/CE Lazio 1, approval date 12/10/2020) |
| Authorization of the Ethics Committee of Roma 6 “Comitato Etico Lazio 2” (protocol number 0216084/2020, approval date 16/12/2020) |
| Authorization of the Ethics Committee of Salerno “Comitato Etico Inter-aziendale Campania Sud” (protocol number 64, approval date 03/11/2020) |
| Authorization of the Ethics Committee of Serenissima “Comitato Etico per la Sperimentazione Clinica della provincia di Venezia e IRCCS S. Camillo (approval date 03/11/2020)28/07/2020) |
| Authorization of the Ethics Committee of Taranto “Comitato Indipendente di Etica Medica” (protocol number 48144, approval date 28/05/2021) |
| Authorization of the Ethics Committee of Teramo “Comitato Etico per le province di L’Aquila e Teramo” (protocol number 11, approval date 24/03/2021) |

**Table S2.** List of the most common comorbidities known to be frequently associated with HA and ICD-9-CM codes necessary to identify the correspondent comorbidities in the administrative database [14].

| **Comorbidity** | **Definition** |
| --- | --- |
| Acute coronary syndrome | At least a discharge diagnosis with ICD-9-CM codes: 410-414 |
| Cardiac dysrhythmias | Discharge diagnosis with ICD-9-CM code: 427 |
| Cerebrovascular disease | Discharge diagnosis with ICD-9-CM codes: 430-438 |
| Atherosclerosis and aneurysm | Discharge diagnosis with ICD-9-CM code: 440-442 |
| Other peripheral vascular disease | Discharge diagnosis with ICD-9-CM codes: 443 |
| Atrial fibrillation | At least a discharge diagnosis with ICD-9-CM code: 427.31 |
| Hypertension | At least 2 prescriptions of antihypertensive drugs with ATC codes: C02, C03, C07-09; or at least 1 hospitalization with ICD-9-CM code: 401; or presence of an exemption (code A31) |
| Dyslipidemia | At least 1 hospitalization with a discharge diagnosis with ICD-9-CM codes: 272.0-4; or at least 2 prescriptions of lipid modifying agents with ATC code: C10AA, C10BA, C10AX13, C10AX14; or the presence of an exemption: 025 |
| Arterial or venous embolism and thrombosis | At least a discharge diagnosis with ICD-9-CM codes: 444, 452-453 |
| Osteoporosis | At least a discharge diagnosis for osteoporosis with ICD-9-CM code: 733.0; or for vertebral fractures with ICD-9-CM codes: 805, 806; or for hip fractures with ICD-9-CM codes: 820 with a replacement procedure codes: 79.00, 79.05, 79.10, 79.15, 79.20, 79.25, 79.30, 79.35, 79.40, 79.45, 79.50, 79.55, 81.51, 81.52 |
| Arthropathy | At least a discharge diagnosis with ICD-9-CM codes: 711-713 |
| HIV (human immunodeficiency virus) | At least a discharge diagnosis with ICD-9-CM codes: V08, 795.71, 079.53, 042-044; or at least 1 prescriptions of direct acting antivirals with ATC codes: J05AE, J05AG, J05AJ, J05AR, J05AX; or an exemption (code 020) |
| HCV (hepatitis C virus) | At least a discharge diagnosis with ICD-9-CM codes: 070.41, 070.44, 070.51, 070.54, 070.7; or at least 1 prescriptions of direct acting antivirals with ATC code: J05AP; or an exemption (code 016.070.54) |
| HBV (hepatitis B virus) | At least a discharge diagnosis with ICD-9-CM codes: 070.2, 070.3, 070.42, 070.52; or an exemption codes 016.070.32, 016.070.33 |
